# History of the Epidemic Yellow Fever at New Orleans, Etc.

**Published:** 1854-04

**Authors:** 


### EDITORIAL.

*History of the Epidemic Yellow Fever at New Orleans, La., in* 1853. By E. D. Fenner, M.D., one of the Visiting Physicians to the New Orleans Charity Hospital; member of American Med. Association, &c. &c. New York: Hall, Clayton & Co, Printers, 46 Pine St., 1854.

This is a Monograph of 84 pages; embracing a pretty full historical, statistical, sanitary, and medical record of that exceedingly fatal epidemic of Yellow Fever which scourged New Orleans and other towns on the Gulf coast and lower Mississippi so severely during the summer and autumn of 1853. The value of the paper is also enhanced by the addition of a few pages containing the conclusions arrived at in the "Second Report of the General Board of Health of England on *Quarantine* in Yellow Fever."—

Dr. Fenner in the work before us gives a very full and exceedingly interesting account of the rise, progress, and termination of the epidemic in the city of New Orleans. He gives an equally frank and full statement of the sanitary condition of the city at the time, showing the local causes of disease to have been most shamefully abundant. He also gives the meteorological and climatic features of the season. But we have only time now to state his conclusions in reference to those points which are of great interest, not only to the profession, but equally so to the municipal authorities of all cities and towns. The Report of the General Board of Health of England was signed by the Earl of Shaftsbury, Mr. Edwin Chadwick, and Dr. T. Southwood Smith. Dr. Fenner remarks: "I must say that my humble observations fully confirm the remarks and conclusions of this learned and able commission." Their conclusions he gives as follows, viz.:—

"From a consideration of the whole of the preceding evidence respecting Yellow Fever, we have arrived at the following conclusions:—

"1. That Yellow Fever Epidemics break out simultaneously in different and distant towns, and in different and distant parts of the same town, often under circumstances in which communication with infected persons is impossible.

"2. That Yellow Fever Epidemics are usually preceded by the occurrence of individual or sporadic cases of the disease, which sporadic cases are likewise common in seasons when no Epidemic prevails.

"3. The Yellow Fever Epidemics, though occasionally extending over large tracts of country, are more frequently limited as to the space over which they spread, often not involving the whole of a town, and sometimes not even any considerable district of it.

"4. That Yellow Fever Epidemics do not spread from district to district by any rule of gradual progression, but often ravage certain localities, while they spare entirely, or visit very lightly, others in the immediate neighborhood, with which the inhabitants are in constant intercommunication.

"5. That Yellow Fever Epidemics, when they invade a district, do not spread from the houses first infected to the next, and thence to the adjoining, and thus extend as from a centre; but, on the contrary, are often confined to particular houses in a street, to to particular houses on one side of a street, to particular rooms in the same house, and often even to particular rooms on the same story.

"6. That in general, when Yellow Fever breaks out in a family, only one or two individuals are attacked; commonly the attendants on the sick escape; and when several members of a family are successively attacked, or the attendants on the sick suffer, either the epidemic was general in the locality, or the individuals attacked had gone into an infected district

"7. That when Yellow Fever is prevalent in a locality, the most rigid seclusion in that locality affords no protection from the disease.

"8. That, on the other hand, so great is the success attending the removal from an infected locality, and the dispersion of the sick in a healthy district, that by this measure alone the further progress of an epidemic is often arrested at once.

"8. That such dispersion of the sick is followed by no transmission of the disease, not even when the sick are placed in the wards of a hospital among patients laboring under other maladies.

"10. That no one of the preceding facts can be reconciled with any other conclusion than that, whatever may be the exciting cause of Yellow Fever, it is local or endemic in its origin: and the evidence of this conclusion is, therefore, cumulative.

"11. That the conditions which influence the localization of Yellow Fever are known, definite, and, to a great exten removable; and are substantially the same as the localizing causes of Cholera, and of all other Epidemic diseases.

"12. That, as in the case of all other Epidemic diseases, in proportion as these localizing causes are removed or diminished, Yellow Fever ceases to appear, or recurs at more distant intervals, and in milder forms.

"13. That besides the common external localizing causes, there is one constitutional predisposing cause of paramount importance, namely, non-acclimatization—that is, the state of the system produced by residence in a cold climate; in other words, European blood exposed to the action of tropical heat; the practical lesson being, that the utmost care should be taken to prevent individuals or bodies of men recently arrived within the Yellow Fever zone, from going into a district in which the disease actually exists, or has recently been present.

"14. That there is no evidence to prove that Yellow Fever has ever been imported.

"15 That consequently the means of protection from Yellow Fever are not quarantine restriction and sanitary cordons, but sanitary works and operations, having for their object the removal and prevention of the several localizing conditions, and when such permanent works are impracticable, the temporary removal, as far as may be possible, of the population from the infected localities.

"We deem it our duty to state, in conclusion, that from the most careful examination which we have been able to make of the mass of evidence submitted to us from which the foregoing conclusions have been deduced, we have not found a single fact or observation clearly ascertained and authentically recorded, opposed to the general tenor of such evidence. We have met with no exceptional cases. We have indeed found the opinions of some authorities, for whom we entertain great respect, not in accordance on some points; but these have reference for the most part to matters of a purely professional and scientific nature. On the great practical question, whether, whatever may be the nature and mode of propagation of Yellow Fever, Quarantine and Sanitary Cordons can afford any real protection against its introduction and spread, we believe there is now a very general unanimity of opinion, in accordance with the evidence we have submitted, that they cannot. We believe there is the like general agreement in this further practical conclusion, that the substitution of Sanitary or Hygienic measures for Quarantine isolation and restriction, would afford more certain and effectual protection."

